# Prognostic implication of novel immune-related signature in breast cancer

**DOI:** 10.1097/MD.0000000000037065

**Published:** 2024-02-09

**Authors:** Bingfeng Chen, Haoming Wu, Yutong Fang, Guangsheng Huang, Cuiping Guo, Chunfa Chen, Lifang He, Zexiao Chen, Xiangling Hou, Cheukfai Li, Jundong Wu

**Affiliations:** a The Breast Center, Cancer Hospital of Shantou University Medical College, Shantou, Guangdong, P.R. China; b Faculty of Science and Technology, BNU-HKBU United International College, Zhuhai, Guangdong Province, China; c Department of Breast Cancer, Guangdong Provincial People’s Hospital, Guangdong Academy of Medical Sciences, Guangzhou, Guangdong Province, China.

**Keywords:** breast cancer, checkpoint inhibitor, prognosis, survival

## Abstract

Checkpoint inhibitor therapy has become increasingly important and has been endorsed as a treatment regimen in breast cancer. But benefits were limited to a small proportion of patients. We aimed to develop an improved signature on the basis of immune genes for detection of potential benefit from immunotherapy. Gene expression data of patients with breast cancer initially extracted from The Cancer Genome Atlas were analyzed. Ten genes were selected from the interaction of differentially expressed genes as well as immune-related genes to develop a survival signature. We compared the high-risk and low-risk groups by gene set enrichment analysis, immune infiltration, checkpoint molecule expression and immunophenoscore. Ten genes were extracted from interactions of differentially expressed and immune-related genes. The immune risk score was determined on the basis of the Cox regression coefficient of hub genes and validated with the GSE96058 dataset. Immune cell infiltrates, including CD8 + T cells, plasma cells, follicular helper T cells, CD4 + memory T cells, M1 macrophages, regulatory T cells and resting NK cells, were more highly infiltrated in the high-risk group as compared to the low-risk group. Checkpoint molecules, including CTLA-4, PD-L1, TIM-3, VISTA, ICOS, PD-1, and PD-L2, were expressed at markedly lower levels in the high-risk group as compared to the low-risk group. Immunophenoscores, as a surrogate of response to immune checkpoint therapy, was observed significant lower in the high-risk group. The 10-gene prognostic signature could identify patients’ survival and was correlated with the biomarkers of immune checkpoint inhibitor therapy, which may guide precise therapeutic decisions in clinical practice.

## 1. Introduction

Worldwide, breast cancer is recognized as the most commonly diagnosed cancer and is the second leading cause of cancer-related deaths among women, thus representing a significant public health concern. In 2017, the United States reported over 250,000 new cases of breast cancer in women.^[1]^ According to the cancer statistics of the National Cancer Institute, 12.8% of women will develop breast cancer throughout their lives. The elevated mortality associated with breast cancer is primarily attributed to resistance to conventional therapeutic regimens and the recurrence of the disease with distant metastasis. Despite significant strides made in clinical research to introduce novel treatment alternatives, the goal of enhancing clinical outcomes remains a pressing challenge,^[2,3]^ the molecular underpinnings and efficacious interventions for advanced breast cancer continue to elude full understanding and require further elucidation. Despite the fact that the multiple gene expression pattern in breast cancer has been thoroughly demonstrated,^[2]^ immune-related gene pattern remains to be studied. A more comprehensive understanding of immune-related gene regulation may reveal the insights of cancer treatment.

Immune checkpoint inhibitors (ICIs), by blockade immune suppressor components including programmed cell death 1 (PD-1), programmed cell death-ligand 1 (PD-L1), and cytotoxic T lymphocyte antigen 4, have shown antitumor activity in multiple types of cancer.^[4]^ Despite ICIs exhibiting a reduced response rate in breast cancer compared to other malignancies such as lung cancer, melanoma, and head and neck cancer, recent updates from numerous clinical trials suggest that their clinical efficacy may be augmented when used in conjunction with chemotherapy. The combination therapy of atezolizumab and nab-paclitaxel, which received FDA approval in the United States, has extended the progression-free survival of patients with advanced triple-negative breast cancer (TNBC) in both the PD-L1-positive cohort and the intention-to-treat population, as demonstrated in the Impassion130 trial.^[1,5,6]^ Pembrolizumab plus chemotherapy was also recently approved for metastatic breast cancer. Preliminary results in neoadjuvant therapy showed a significant enhance of the pathological complete response rate (*P* = .00055) versus placebo, and the results were also independent of PDL1 + status.^[7,8]^

Most breast cancers have low PD-L1 expression and a low mutation burden, resulting in difficulty in selecting patients to receive ICIs. Several immune-related prognostic biomarker models have been reported to evaluate immune-related survival in breast cancer, especially in TNBC.^[3,9,10]^ However, few studies have shown a model with the potential to detect patients who should receive ICIs. To select a precise treatment option in breast cancer immunotherapy, it is vital to establish a multi-immune gene-relevant prediction model for ICI responsiveness.

In the present study, we conducted an analysis of the cancer genome atlas (TCGA) database to explore immune-related gene sets and those associated with survival in breast cancer. Subsequently, we constructed a prognostic prediction signature. Moreover, we evaluated this signature by examining immune cell infiltration, the mutational landscape, the expression of checkpoint inhibitors, and immunophenotypic scores to identify breast cancer patients who are most likely to derive clinical benefit from treatment with immune checkpoint inhibitors.

## 2. Materials and methods

### 2.1. Patients and data acquisition

Relevant clinical data as well as RNA-sequencing data of breast cancer samples were obtained from UCSC Xena (http://xenabrowser.net). A list of immune-related genes (IRGs) was identified and further downloaded from the Immunology Database and Analysis Portal (ImmPort). Moreover, we acquired a list of transcription factor data from the Cistrome Cancer database.

### 2.2. Differential gene analysis

To identify genes potentially implicated in the pathogenesis or progression of breast cancer, we analyzed differentially expressed genes (DEGs) between tumor and adjacent normal tissues using the limma package. Identification of DEGs was performed, with an adjusted *P* value of <.05 and fold change (FC) > 1.6 or < 0.667. Differentially expressed immune-related genes (DE-IRGs) screened by the cutoff criteria were extracted from the intersection between the list of IRGs and the list of DEGs.

### 2.3. Functional enrichment analysis

For the assessment of the underlying mechanisms of DE-IRGs, functional enrichment analysis was performed on the basis of Gene Ontology and Kyoto Encyclopedia of Genes and Genomes with the use of the clusterProfiler package of R software.

### 2.4. Molecular characteristics of hub IRGs

Clinical characteristics and pathologic factors of breast cancer patients and copy number alteration data were downloaded from cBioPortal^[11]^ (http://cbioportal.org). Based on the Search Tool for the Retrieval of Interacting Genes/Proteins (STRING) database (http://string-db.org), the protein–protein interaction network of IRGs was constructed. Transcription factor data were extracted from the Cistrome Cancer database^[12]^ (http://cistrome.org/CistromeCancer). MuTect2 analysis was used to illustrate somatic mutations.

### 2.5. Development and validation of the immune signature

The TCGA-BRCA patient cohort was used to develop the immune signature of the breast cancer prognostic immune gene risk model. Univariate Cox proportion hazard regression was performed for the investigation of the prognostic effect of hub IRGs. This risk score was determined for individual patient according to a linear combination of expression weighted by the Cox regression coefficient of hub IRGs. The immune-related risk score of the signature was determined with the use of the following formula: Risk score = coefficient of Gene a* expression level of Gene a + coefficient of Gene b* expression level of Gene b+… coefficient of Gene n* expression level of Gene n. According to the risk signature (median cutoff value), patients were further stratified into high-risk and low-risk groups. The Kaplan–Meier method was then applied for survival to validate the prognostic effect of the risk score.

### 2.6. Immune infiltration analysis and expression of immune checkpoint inhibitors

Utilizing CIBERSORT (http://cibersort.stanford.edu),^[13]^ we leveraged TCGA gene expression data to quantify the proportions of 22 immune cell subtypes infiltrating the breast cancer microenvironment. The comparison of immune cell infiltration between the high- and low-risk groups was performed by using a two-sided Wilcoxon rank-sum test. The mRNA levels of several immune checkpoint molecules, including PD-1, PD-L1, VISTA, TIM-3, CTLA-4, PD-L2, and ICOS, were compared between the 2 groups.

### 2.7. Gene set enrichment analysis

Gene set enrichment analysis (GSEA) was conducted in this study with the TCGA-BRCA dataset using GSEA v4.0.1 software (Broad Institute).^[14,15]^ Assessment of patients was performed and they were further classified into a high-risk group and a low-risk group. The enrichment of high-risk and low-risk gene sets was computed in the form of “‘high’” versus ‘‘low’’, with a permutation number of 1000. Hallmark and immune signature gene set databases were downloaded through GSEA software. Gene sets were processed to be significantly enriched with a false discovery rate (FDR) of <0.25 and *P* value of <.05.

### 2.8. Immunophenoscore

Immunophenoscore (IPS) was determined by machine learning identification of the scoring scheme for quantification as the predictor for response to immunotherapy, including anti-CTLA-4 and anti-PD-1.^[16,17]^ IPS was graded with a quantitative score from 0 to 10 representing tumor immunogenicity. IPS data were extracted from The Cancer Immunome Atlas (TCIA, https://tcia.at).

### 2.9. Statistical analyses

Statistical analyses were conducted using R software (version 3.5.2, R Foundation for Statistical Computing, Vienna, Austria). Additionally, gene functional enrichment analyses were executed utilizing the clusterProfiler package within R. Moreover, we calculated the AUC of the survival ROC curve via the survival ROC package in R software. Pearson chi-squared, Fisher exact test, or the Kruskal–Wallis test were selected to identify the potential differences among the variables. Continuous variables were further compared via the Mann–Whitney U test or Student *t* test. The compared categorical data were based on the chi-squared test. *P* values were two-tailed, and statistically significant differences were determined when the *P* value was <.05. Survival analysis was conducted on the basis of the Kaplan–Meier method, and 2 groups were compared based on the log-rank test. Cox proportion hazard regression analysis was conducted with patient survival.

## 3. Results

### 3.1. Identification of DE-IRGs

**Figure 1.**
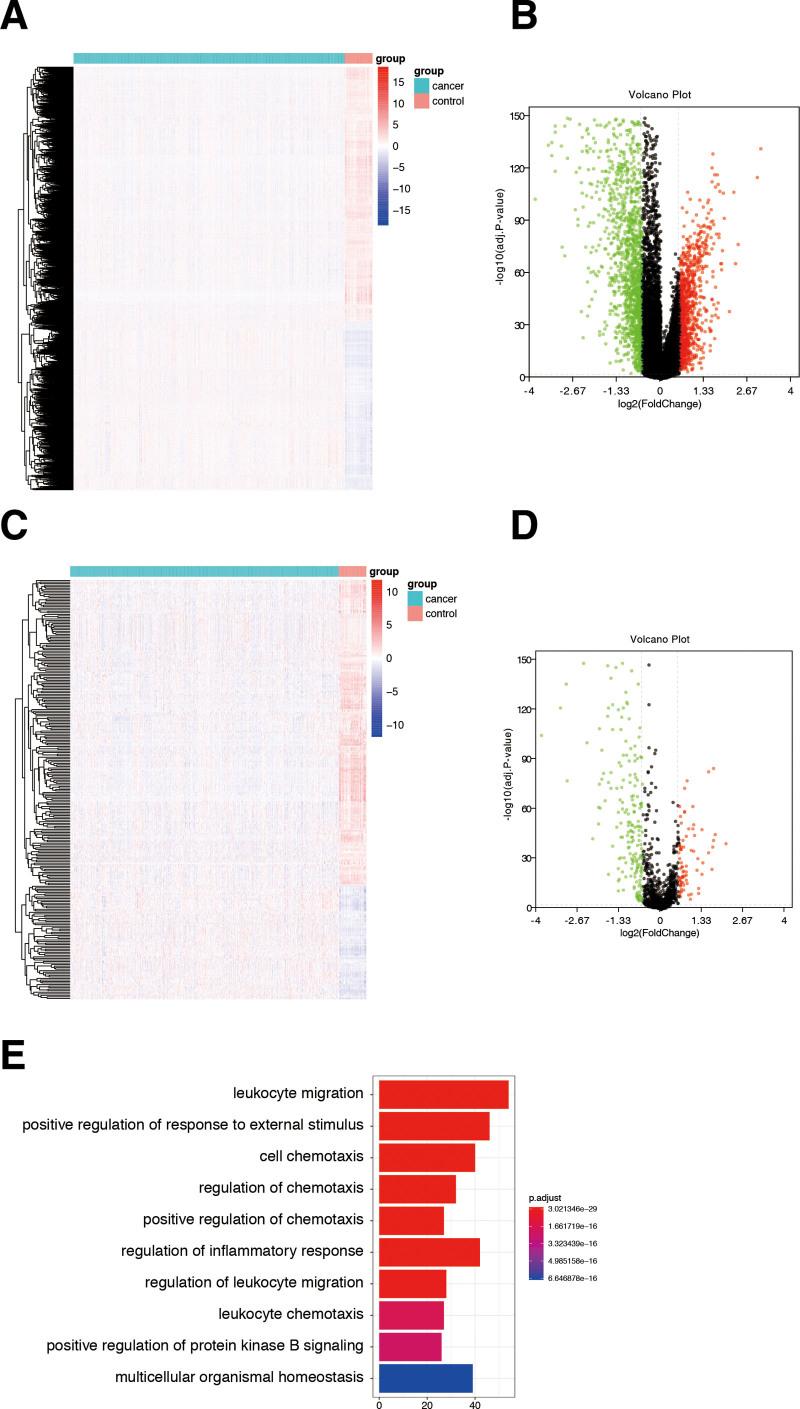
Identification of DEGs and IRGs. (A) Heatmap of DEGs in breast cancer patients from the TCGA cohort. (B) Volcano plot of DEGs in breast cancer patients from the TCGA cohort. (C) Heatmap of DE-IRGs in breast cancer in the TCGA cohort. (D) Volcano plot of DE-IRGs in breast cancer patients from the TCGA cohort. (E) Enrichment analysis of the DE-IRGs. DEGs = differentially expressed genes, DE-IRGs = differentially expressed immune-related genes, IRGs = immune-related genes, TCGA = the cancer genome atlas.

RNA-sequencing data and clinical characteristics of the TCGA dataset of 1104 breast cancer samples were used for this analysis. A total of 2395 DEGs were screened out, including 946 upregulated genes and 1449 downregulated genes (Fig. 1A and B). We proceeded with the DEGs set with interactions with the list of IRGs list derived from ImmPort. The interactions of 273 DE-IRGs were retrieved, including 74 upregulated IRGs and 199 downregulated IRGs (Fig. 1C and D). Enrichment analyses were performed, revealing that the DE-IRGs had high association with leukocyte migration, positive regulation of response to external stimulus, cell chemotaxis and regulation of leukocyte migration (Fig. 1E). In Kyoto Encyclopedia of Genes and Genomes analysis, the 4 most highly enriched pathways were the cytokine-cytokine receptor interaction, PI3K-Akt signaling pathway, MAP signaling pathway and chemokine signaling pathway.

### 3.2. Identification of survival-associated DE-IRGs and hub IRGs

**Figure 2.**
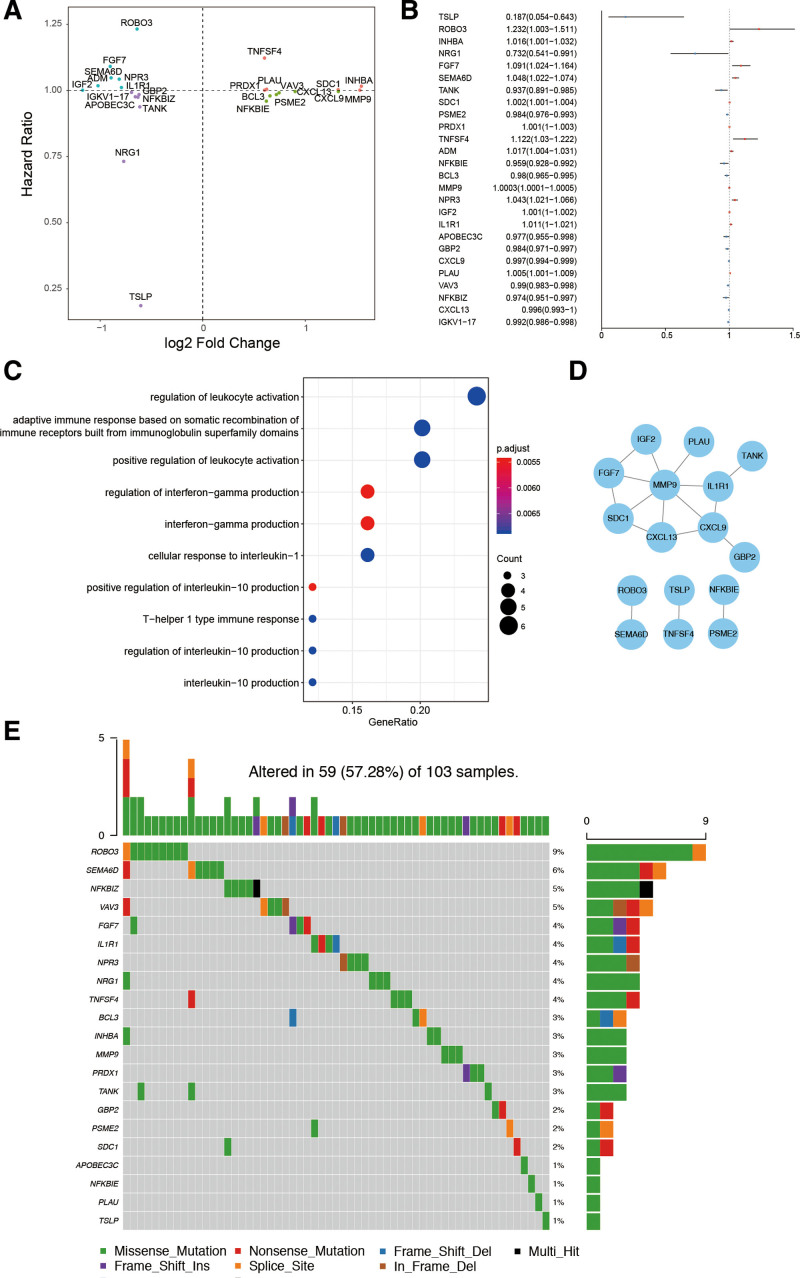
Identification of survival-associated DE-IRGs. (A) Scatter plot of hazard ratios of survival-correlated DE-IRGs. (B) Forest plot of hazard ratios of survival-correlated DE-IRGs. (C) Enrichment analysis of 26 hub DE-IRGs. (D) Protein–protein interaction network of the 26 genes. (E) Somatic mutation profile of the 26 hub genes. DE-IRGs = differentially expressed immune-related genes.

The above 273 DE-IRGs were used to uncover prognostic factors in breast cancer survival. 26 DE-IRGs were correlated with breast cancer survival in univariate Cox regression analysis and were regarded as hub genes (Fig. 2A). Thirteen genes were identified as protective factors, and 13 were risk factors (Fig. 2B). Enrichment analyses were also performed with using the 26 hub DE-IRGs. Among them, the most highly enriched pathways were the regulation of leukocyte activation (Fig. 2C). The protein-protein interaction network showed that MMP9 and CXCL9 were key hub genes in 26 hub genes (Fig. 2D). The mutation profile of 26 hub DE-IRGs is illustrated (Fig. 2E). TOBO3 had the highest mutation frequency among these mutated genes.

### 3.3. Transcription factor network of hub IRGs

**Figure 3.**
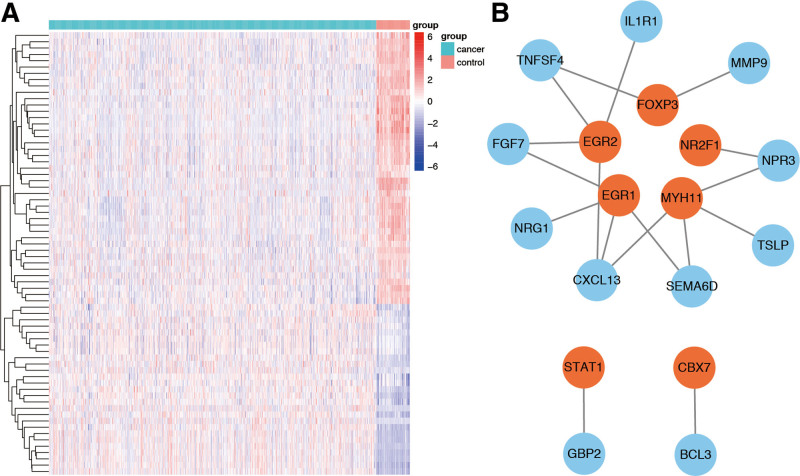
Identification of DE-TFs. (A) Heatmap of DE-TFs. (B) Regulatory network of transcription factors and the 26 hub IRGs. DE-TFs = differentially expressed TFs, IRGs = immune-related genes.

In order to explore the potential biobehavior of hub DE-IRGs, we performed transcription factor analysis of 26 hub DE-IRGs. Among the 318 transcription factors (TFs) derived from the Cistrome Cancer database, 70 were differentially expressed TFs (DE-TFs, Fig. 3A). Among these 70 DE-TFs, 37 TFs were associated with breast cancer survival. Using the 37 TFs and 26 hub DE-IRGs, a regulatory network was constructed. A correlation value >0.4 with a combined score >0.6 was applied as the cutoff value and considered significant. The regulatory network indicated the relationship between TFs and hub DE-IRGs, showing a potential regulatory mapping of MYH1, EGR1, EGR2, and FOXP3 (Fig. 3B).

### 3.4. Development of the immune-related risk signature

**Figure 4.**
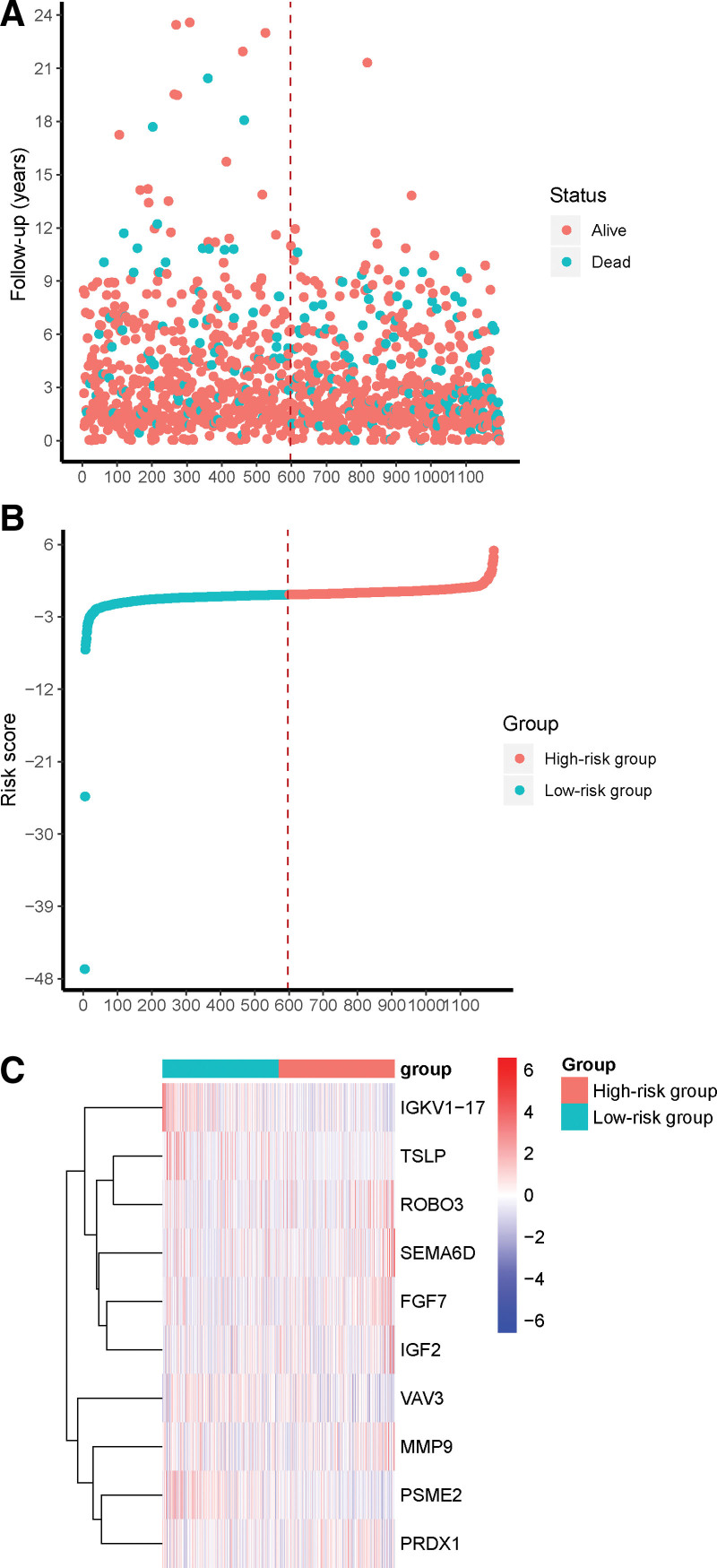
Development of immune-related prognostic signature. (A) Follow-up years according to the immune-related prognostic signature. (B) High-risk and low-risk classification according to the immune-related prognostic signature. (C) Heatmap of the 10 DEGs between the high-risk and low-risk groups. DEGs = differentially expressed genes.

**Figure 5.**
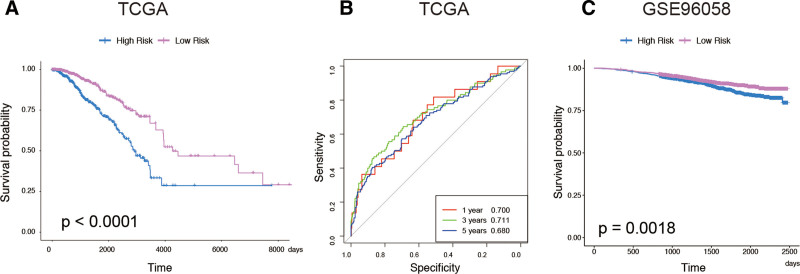
Assessment of the immune-related prognostic signature. (A) Kaplan–Meier curve analysis of OS between the high- and low-risk groups in the TCGA. (B) ROC curves of the 1-, 3-, 5-year survival in the TCGA. (C) Kaplan–Meier curve analysis of OS between the high- and low-risk groups in the GSE96058 dataset. TCGA = the cancer genome atlas

For the determination of prognostic value of the above 26 DE-IRGs that were associated with breast cancer survival, an immune-related risk signature was established with DE-IRGs. After screening by multivariate Cox regression analysis, 10 DE-IRGs were selected as independent survival biomarkers of breast cancer and were used for model development. The predicted signature was defined according to the linear-combination-based model. The formula combined the expression levels of 10 DE-IRGs weighted by the corresponding coefficient in multivariate Cox regression analysis as follows: risk score = (−1.67518573* expression of *TSLP*) + (0.351942883* expression of *ROBO3*) + (0.114943322* expression of *FGF7*) + (0.069135618* expression of *SEMA6D*) + (−0.014734708* expression of *PSME2*) + (0.001990887* expression of *PRDX1*) + (0.000255536* expression of *MMP9*) + (0.000981797* expression of *IGF2*) + (−0.010857713* expression of *VAV3*) + (−0.006919455* expression of *IGKV1-17*). All patients were calculated with the risk score (Fig. 4A). According to the median value as the optimal cutoff (−0.2565), patients were then divided into a high-risk group and a low-risk group (Fig. 4B). A heatmap of the expression of DE-IRGs revealed distinct expression patterns between the high-risk group and the low-risk group (Fig. 4C). The high-risk patients showed significantly lower overall survival (OS) than low-risk patients (Fig. 5A, *P* < .0001). The 1-year and 5-year AUC of the survival ROC curve were respectively 0.700 and 0.711 (Fig. 5B). To further test the robustness of the risk model, we performed an external validation with the GSE96058 dataset. Accordingly, high-risk patients had a lower OS as compared to low-risk patients (Fig. 5C, *P* = .0018).

### 3.5. The immune-related risk signature was associated with the immune infiltration pattern, GSEA function and clinicopathologic features

**Figure 6.**
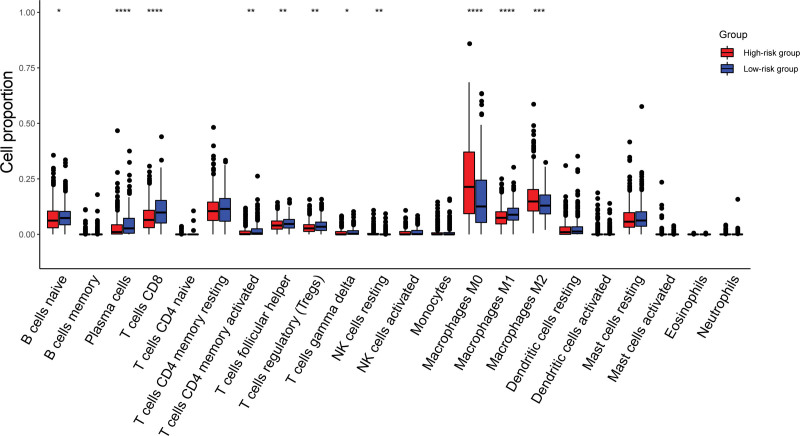
Comparison of the tumor-infiltrating immune cells according to the immune-related risk signature.

To investigate which subset of immune cells may be correlated with the immune-related risk signature, the immune cell infiltration proportions of high-risk and low-risk patients were determined (Fig. 6A). CD8 + T cells, plasma cells, M1 macrophages, follicular helper T cells, CD4 + memory T cells, resting NK cells and regulatory T cells were more highly infiltrated in samples of the low-risk group, while M0 macrophages and M2 macrophages were more highly infiltrated in the high-risk group.

**Figure 7.**
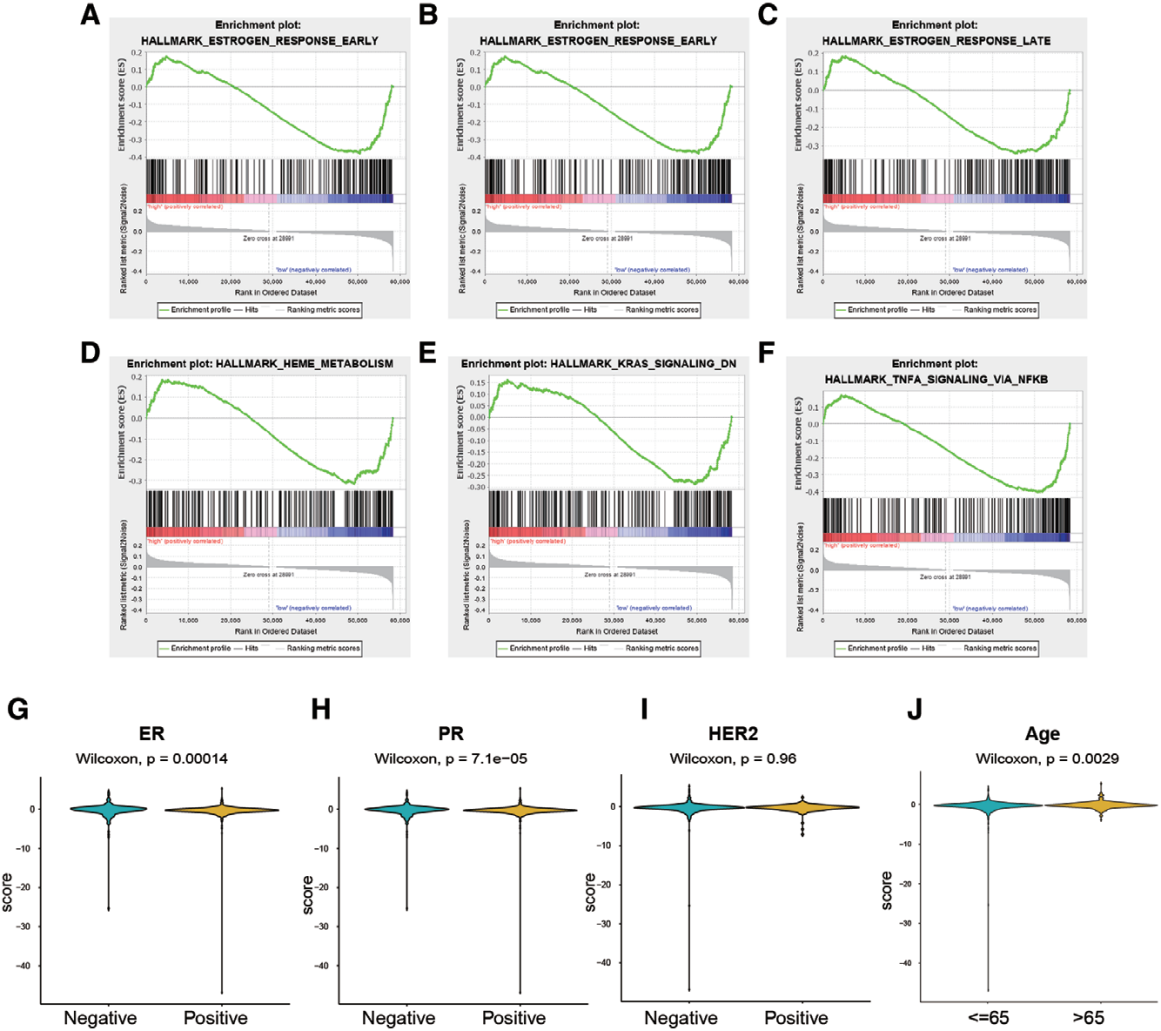
GSEA results (A–F) and clinicopathological factors (G–J) between the high-risk and low-risk patients. GSEA = gene set enrichment analysis.

GSEA revealed that the immune-related high-risk group was significantly enriched in estrogen response, HEME metabolism, KRAS signaling and TNF-α signaling (Fig. 7A–F).

We also analyzed the association between clinical factors and the immune-related risk signature. In the ER-positive and PR-positive groups, immune-related risk scores were significantly higher than those in the ER-negative and PR-negative groups, respectively (Fig. 7G and H). However, there was no statistical difference in risk scores between the HER2-positive and HER2-negative groups (Fig. 7I). A higher risk score was observed in patients aged ≥65 (Fig. 7J), while no differences were observed when comparing the high- and low-risk patients in TNM stage.

### 3.6. Immune-related risk signature was associated with ICI therapy

**Figure 8.**
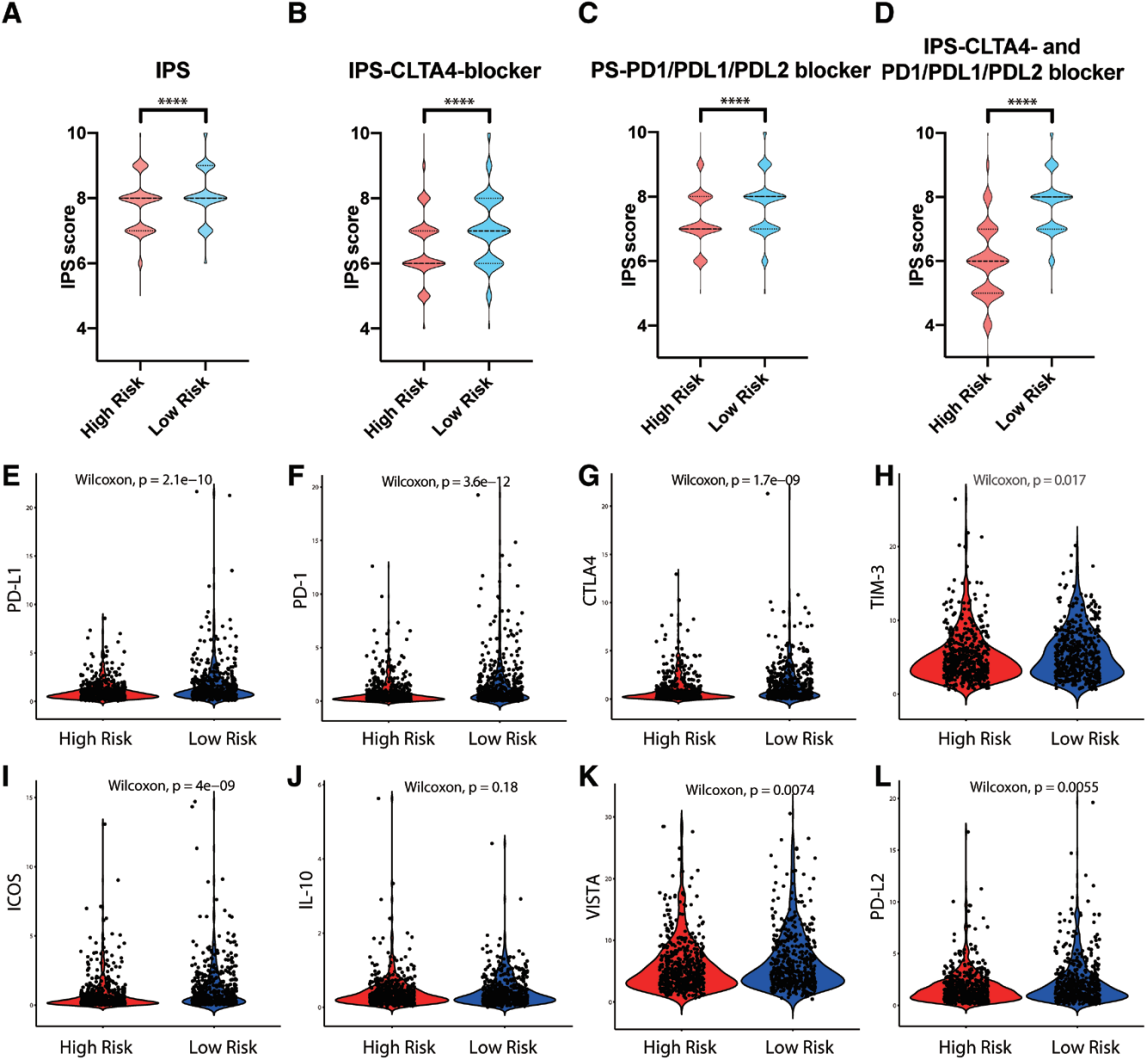
Immune checkpoint expression and immunophenoscore according to the immune-related risk signature. (A–H) The association between immune checkpoint molecule expression and the immune-related risk signature in breast cancer patients. (I–L) The association between the immunophenoscore and immune-related risk signature in breast cancer patients.

A previous study reported the IPS as a predictor of the response to ICI therapy, which was constructed based on 4 gene categories: effector cells, suppressive cells, MHC-related molecules and immunomodulators.^[16]^ Since the cohort with ICI therapy in breast cancer was not available, we used IPS as a surrogate of the breast cancer patients’ response to ICI. In the immune-related signature high-risk group, the IPS was significantly higher in the 4 groups and included IPS, IPS-CLTA4 blocker, IPS-PD1/PDL1/PDL2 blocker, IPS-CLTA4- and PD1/PDL1/PDL2 blocker (Fig. 8A–D, *P* < .0001). Moreover, we also compared the mRNA expression of checkpoint inhibitors and the ligands between the high-risk and low-risk groups. A lower expression of PD-L1, VISTA, TIM-3, CTLA-4, ICOS, PD-1, and PD-L2 was observed in the high-risk group when comparing the low-risk group (Fig. 8E–L).

## 4. Discussion

In this study, we established a prognostic signature based on immune genes to predict survival outcomes, leveraging data from the breast cancer cohort within the TCGA database. Breast cancer, characterized by its heterogeneity, is typically categorized by the status of estrogen receptor, progesterone receptor, and HER2 expression. The early usage of multigene panels, such as OncoType DX^[18]^ and MammaPrint,^[19]^ was established to guide chemotherapy options. However, they were mainly for HR+/HER2− disease. Other than those reported genes in the 21-gene and 70-gene panels, immune-related genes were rarely reported in prognostic models. After evaluation by multivariate Cox regression analysis, 10 genes were selected as independent survival biomarkers of breast cancer. According to the immune-related gene signature, patients were classified into high-risk and low-risk groups. In a recent study, we found that poorer survival was found among patients in the high-risk group compared to those in the low-risk group. The predictive value remained robust in the validation cohort. We suggest a more careful and frequent follow-up plan for high-risk patients.

Prior research has indicated that certain genes are implicated in the formation and control of the tumor microenvironment. For example, thymic stromal lymphopoietin (TSLP), known for fostering an inflammatory Th2 microenvironment, has been documented to inhibit the progression of breast cancer tumors.^[20]^ Indeed, TSLP has the potential to suppress inflammation in para-tumor tissue in early-stage breast cancer.^[21]^ However, in metastatic disease, TSLP is essential for breast cancer progression through the induction of the anti-apoptosis molecule Bcl-2 in vitro.^[22]^ In our 10-gene signature, TSLP had the most protective effect on OS. Some of the other genes in this 10-gene signature have rarely been reported in breast cancer, and their biological functions remain to be further elucidated.

The clinical practices of Prosiga Prediction Analysis of Microarray 50 (PAM50), EndoPredict, and Breast Cancer Index^[23]^ are widely acknowledged. Recently, other new prognostic signatures in breast cancer were constructed. Xie et al reported a 12-gene prognostic signature for predicting clinical outcomes.^[24]^ Another study conducted by Jiang et al established a prediction model based on immune cell subsets for immunotyping and individualized treatment.^[25]^ Although some other gene signatures have been developed,^[26–28]^ immune-related gene signatures related to immune checkpoint inhibition are still absent.

Based on earlier studies, the application value of ICI therapy in the treatment of breast cancer is very limited. As our understanding of the molecular subtypes and immune system of breast cancer has improved, immunotherapy has therapeutic value for a portion of breast cancer subtypes. In some aggressive breast cancer subtypes, including TNBC and HER2-positive breast cancer, tumor-infiltrating lymphocytes play an important role in preventing tumor growth, which is important for both early-stage and advanced breast cancer. Large-scale studies have found that more tumor-infiltrating lymphocytes are linked to prolonged survival of patients with early TNBC^[29]^ and metastatic HER2-positive breast cancer.^[30]^ The disappointing response rate is not more than 22% with immune monotherapy,^[31,32]^ but in recent trials, chemotherapy has been added to improve the response to immunotherapy in some populations of breast cancer patients. In other words, a more specific predictor is needed to better select the group of patients who may benefit from immunotherapy regimens in breast cancer.

To date, the PD-L1 expression status is considered the recommended selection criterion for selecting patients to receive ICIs. The immune cell PD-L1 status evaluated by SP142 is 41% positive in patients in the Impassion130 trial.^[6]^ Among patients in the SP142-positive subgroup, atezolizumab prolonged the median OS by 7 months in advanced TNBC. In the KEYNOTE-522 trial, PD-L1 expression was assessed with the application of the 22C3 assay.^[7]^ If antibodies are used to test the same specimens in an assay, such as the SP263 or 22C3 assay, there will be more positive patients than those tested using the SP142 assay. However, assays other than the SP142 assay fails to predict the efficacy of atezolizumab in metastatic breast cancer, which may cause overtreatment. Thus, different detection assays particularly affect the predictive value. Moreover, PD-L1 expression may also be affected by tumor heterogeneity and may change dynamically during treatment. Of note, single selection criteria utilizing PD-L1 expression as a gold standard remain uncertain.

Since PD-L1 expression suffer from specific limitations, we developed the current immune-related gene signature that may be associated with immunotherapy. Although these 10 genes were not the most prognostic genes, the genes we included were all immune-related genes. Therefore, the 10-immune-related gene signature was not only an effective tool in terms of predicting prognosis but also a model to detect the implication of immune checkpoint therapy. According to GSEA and pathway analysis, these 10 genes were found to be mainly related to cytokine-cytokine receptor interactions and immune activation pathways, such as leukocyte activation, interferon-gamma production, and Th1 immune response. When referring to clinicopathological factors, the immune-related risk signature was not related to staging and PAM50 classification but was related to age, ER status and PR status. The immune response represents a complex interplay among various specialized cell subtypes, each acting in a highly orchestrated fashion. To gain deeper insights into the character and heterogeneity of breast tumor immune responses, it is essential to dissect the immune cells with an approach that elucidates their unique functional spectrum. A previous study applied CIBERSORT to establish the relationship between immune infiltrates and molecular subtypes, survival and response to chemotherapy in breast cancer.^[33]^ In the high-risk group, different proportions of infiltration of multiple immune cells were also observed. By evaluating the mRNA expression between immune checkpoint molecules, the high-risk group showed lower PD-L1 expression as compared to the low-risk group, which may result in a lower response to ICIs. Our research suggested that patients with a low risk of 10 IRG markers may have the immunodominant tumor environment. The abovementioned results provided further support for the hypothesis that this immune signature was regarded as a potential model for the determination of breast cancer patients harboring immunodominant tumors with higher incidence of responding to ICIs.

However, the correlation between immune score and ICI response has not been validated in an immunotherapy cohort, so further research is needed.

The present study is subject to several limitations that warrant mention. Firstly, the retrospective nature of the study and the reliance on a public database for developing the prognostic signature may introduce inherent biases. Secondly, the absence of data on responses to checkpoint inhibitors within the breast cancer cohort poses challenges in appraising the true efficacy of immunotherapy. In light of these findings, it is imperative that our results undergo validation through a prospective and more exhaustive investigation.

## 5. Conclusion

This study established a risk signature based on 10 immune-related gene markers in breast cancer, in which high scores were independently related to markedly worse prognosis. The signature can be used as a powerful as well as an accurate tool to predict the survival of breast cancer patients. What’s more, tumors in the high-risk group tended to have lower checkpoint inhibitor expression and IPS score. These findings provide new implication of ICI therapy in breast cancer but require prospective validation.

## Author contributions

**Conceptualization:** Jundong Wu, Bingfeng Chen, Yutong Fang, Chunfa Chen, Xiangling Hou, Cheukfa Li.

**Data curation:** Jundong Wu, Yutong Fang, Guangsheng Huang, Chunfa Chen, Cheukfa Li.

**Formal analysis:** Jundong Wu, Yutong Fang.

**Funding acquisition:** Cuiping Guo, Lifang He, Zexiao Chen.

**Investigation:** Bingfeng Chen, Haoming Wu, Cuiping Guo, Lifang He.

**Methodology:** Haoming Wu.

**Resources:** Cuiping Guo.

**Software:** Guangsheng Huang, Zexiao Chen.

**Supervision:** Bingfeng Chen, Haoming Wu.

**Validation:** Bingfeng Chen.

**Writing – original draft:** Bingfeng Chen, Yutong Fang.
